# Emergent role of gasotransmitters in ischemia-reperfusion injury

**DOI:** 10.1186/2045-9912-1-3

**Published:** 2011-04-27

**Authors:** Bridgette F Moody, John W Calvert

**Affiliations:** 1 Department of Surgery, Division of Cardiothoracic Surgery, Carlyle Fraser Heart Center, Emory University School of Medicine, Atlanta, GA 30308, USA

**Keywords:** Nitric oxide, carbon monoxide, hydrogen sulfide, cytoprotection, ischemia-reperfusion injury

## Abstract

Nitric oxide (NO), carbon monoxide (CO) and hydrogen sulfide (H_2_S) are lipid-soluble, endogenously produced gaseous messenger molecules collectively known as gasotransmitters. Over the last several decades, gasotransmitters have emerged as potent cytoprotective mediators in various models of tissue and cellular injury. Specifically, when used at physiological levels, the exogenous and endogenous manipulation of these three gases has been shown to modulate ischemia/reperfusion injury by inducing a number of cytoprotective mechanisms including: induction of vasodilatation, inhibition of apoptosis, modulation of mitochondrial respiration, induction of antioxidants, and inhibition of inflammation. However, while the actions are similar, there are some differences in the mechanisms by which these gasotransmitters induce these effects and the regulatory actions of the enzyme systems can vary depending upon the gas being investigated. Furthermore, there does appear to be some crosstalk between the gases, which can provide synergistic effects and additional regulatory effects. This review article will discuss several models and mechanisms of gas-mediated cytoprotection, as well as provide a brief discussion on the complex interactions between the gasotransmitter systems.

## Introduction

Nitric oxide (NO), carbon monoxide (CO) and hydrogen sulfide (H_2_S) are lipid-soluble, endogenously-produced gaseous messenger molecules [1]. Together, they make up the family of labile biological mediators termed gasotransmitters. Historically, these gases were considered to be highly toxic and hazardous to the environment. However, it was found that under normal physiological conditions in mammals these molecules are enzymatically regulated and endogenously produced. Because of this discovery, the biological and physiological role of these gases has been re-evaluated. As such, an extensive amount of work has been conducted over the last several decades (last three centuries for NO) and has led to the discovery that each gasotransmitter possess a number of physiological actions. The gasotransmitters have also been extensively studied in several models of cellular and tissue injury. This work has led to the discovery that gasotransmitters and the enzymes that generate them share similar features and overlap in a variety of biological functions. Specifically, studies have found that deficiencies in the enzymes (through genetic manipulation or use of inhibitors) exacerbate ischemia-reperfusion (I/R) injury, whereas genetic overexpression of the enzymes induces cytoprotection. Furthermore, treatment with pharmacological donors or inhaled gas therapy has also been shown to provide cytoprotection. This review article will discuss the physiological significance and the fundamental mechanisms by which these gaseous molecules exert cytoprotection in several models of tissue and cellular injury, as well as provide a brief discussion on the complex interactions between the gasotransmitter systems.

### Physiological and Biological Roles of Gasotransmitters

**Figure 1:**
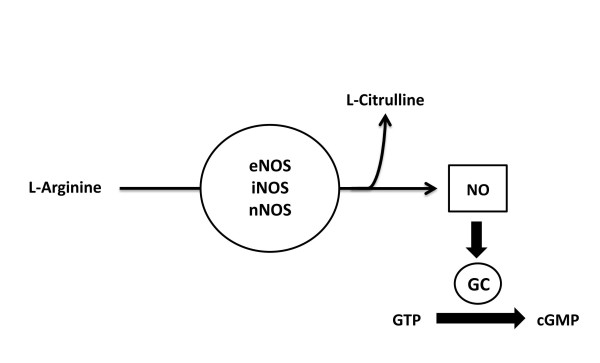
**Enzymatic Synthesis of Nitric Oxide**. Nitric oxide (NO) is produced from amino acid L-arginine by the enzymatic action of nitric oxide synthase (NOS). There are there forms of NOS: endothelial NOS (eNOS), neuronal NOS (nNOS), and inducible NOS (iNOS). NO activates guanylate cyclase (GC), which leads to increased production of 3',5'-monophosphate (cGMP).

NO was the first gasotransmitter to be identified by studies dating back to the late 1700's, which investigated its pharmacological efficacy [2]. However, it was not until 1867 that evidence emerged to suggest that NO induced vasodilatory effects in patients suffering from angina pectoris [3]. Unfortunately, it's true potential and physiological significance in the field of medicine was not discovered until the 1980's, when scientists Furchgott and Zawadzki identified NO as an endogenous modulator of vascular tone [4]. NO levels are controlled at the level of synthesis, initiated by the interaction of nitric oxide synthases (NOSs) and calcium-calmodulin stimulation. There are three isoforms of NOS that have been characterized, purified, and cloned: the endothelial isoform (eNOS), the neuronal isoform (nNOS), and the inducible isoform (iNOS). These enzymes generate NO from the guanidine nitrogen of the amino acid L-arginine in the presence of oxygen and NADPH, while forming L-citrulline as a byproduct (Figure 1). NO released from the endothelium enters the target cell and initiates cGMP-dependent protein kinase phosphorylation of myosin, by activating the cytosolic enzyme soluble guanylyl cylase causing a subsequent increase in the intracellular concentration of cyclic GMP (cGMP), which then goes on to regulate smooth muscle relaxation and vasodilatation.

**Figure 2:**
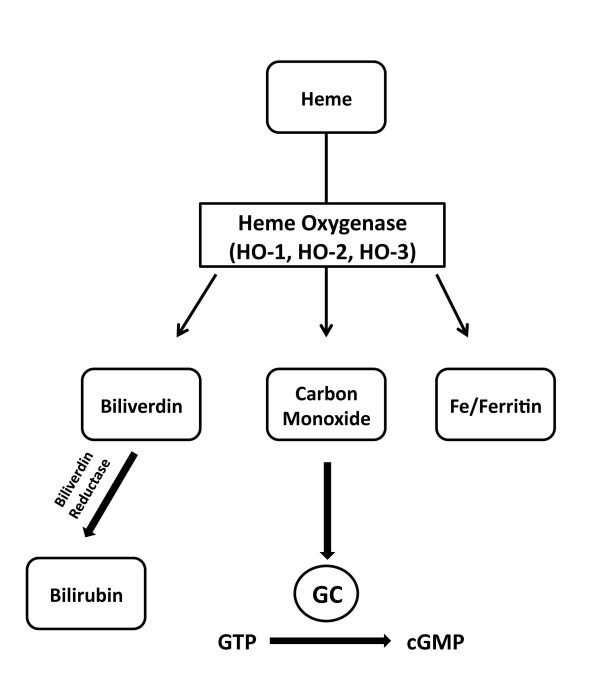
**Enzymatic Synthesis of Carbon Monoxide**. Heme is catabolised by heme oxygenases (HO), to form biliverdin, carbon monoxide, and iron. Carbon monoxide can activate soluble guanylyl cyclase, which causes an increase in cyclic guanosine monophosphate levels (cGMP). Biliverdin is subsequently converted to bilirubin by biliverdin reductase.

The endogenous source of CO was first identified in 1969 when it was determined that it is derived from the breakdown of heme by the enzyme heme oxygenase (HO) (Figure 2) [5]. In 1993, a study investigating nonadrenergic/noncholinergic (NANC) neurotransmission in the enteric nervous system identified CO as a vasorelaxant and later confirmed it as the second gasotransmitter [6,7]. Molecular cloning has revealed three known isoforms of heme oxygenase: inducible HO-1 a ubiquitously expressed transcription factor activator that is crucial in oxidative stress response; HO-2 which is constitutively active and controlled by posttranslational modification [8]; and HO-3 which is similar to HO-2 but considered a less efficient heme catalyst [9]. Similar to NO, CO has been shown to modulate vasorelaxation, vascular smooth muscle cell growth, and tissue injury through elevation of cGMP levels, it is also suggested that local effects of CO may directly influence NO release, and furthermore stimulate adaptive responses and augment gene expression [10,11].

**Figure 3:**
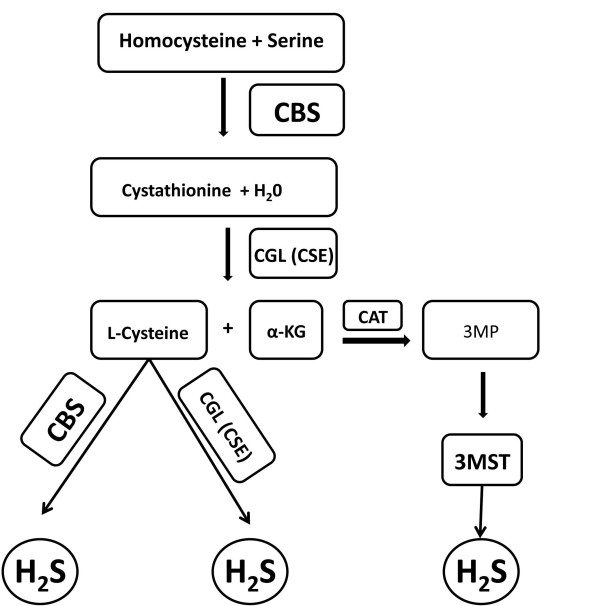
**Enzymatic Synthesis of Hydrogen Sulfide**. There are three enzymatic pathways involved in the synthesis of hydrogen sulfide (H_2_S) in mammalian systems. Cystathionine β-synthase (CBS) produces H_2_S through a reaction involving the generation of cystathionine from homocysteine and L-cysteine from cystathione. Cystathionine γ-lyase (CGL or CSE) produces H_2_S through a reaction involving the generation of L-cysteine from cystathionine. 3-mercaptopyruvate sulfur transferase (3MST) produces H_2_S through a reaction involving the generation of 3-mercaptopyruvate (3MP) from α-ketoglutarate (α-KG) by cysteine aminotransferase (CAT).

H_2_S was the third endogenously produced gasotransmitter to be identified. The production of H_2_S in mammalian systems has been attributed to three principal enzymes (Figure 3): cystathionine β-synthase (CBS), cystathionine γ-lyase (CSE or CGL) and 3-metacaptopyruvate sulfur transferase (3MST). The endogenous production of H_2_S was initially described in the brain and attributed to CBS activity [12]. However, recent studies have found that ~90% of total H_2_S production in the brain is attributed to 3MST [13]. CBS and CGL are found in all tissues; however CBS is the predominant source of H_2_S in the central nervous system (CNS), whereas CGL is the predominant source in the cardiovascular system. Perhaps the most characterized physiological action of H_2_S is its participation in memory formation as a central component of the process of long-term-potentiation of neuronal circuitry [12]. Additionally, like NO and CO, H_2_S also mediates smooth muscle relaxation and vasodilation. However, it does so in a guanylyl cyclase/cGMP independent manner [14,15].

### Cytoprotective Effects of Gasotransmitters

A unique characteristic of gasotransmitters is that they lack conventional regulatory mechanisms, as they have the ability to pass messages directly to an intracellular target without the need for receptor or plasma membrane interactions [16]. This makes the gasotransmitters particularly attractive candidates for the treatment of pathological disorders, such as I/R injury. Over the past several decades, studies using animal models and clinical investigations have defined these gaseous molecules as physiological participants in a wide range of profound biochemical and biological functions, and have defined them as potent cytoprotective mediators in various models of tissue and cellular injury. In the physiological range, the exogenous and endogenous manipulation of these three gases has been shown to modulate ischemia/reperfusion injury, vascular damage, vasodilation, oxidative stress, inflammation, and apoptosis.

### Myocardial Ischemia-Reperfusion Injury

In terms of cytoprotective effects, NO has been the most investigated gasotransmitter. Specifically, much of this work has focused on the role of endogenously and exogenously derived NO in mediating the effects of myocardial I/R injury [17-21]. The role of endogenously derived NO has been studied using pharmacological inhibitors against NOS and by genetically targeting each NOS. The role of exogenously derived NO has been studied through the administration of NO in the form of authentic NO gas, NO donors, and more recently nitrite and nitrate. Perhaps the most clear-cut evidence for a protective role of endogenously derived NO in the setting of myocardial injury comes from studies aimed at investigating eNOS [22]. Studies that have employed the use of mice deficient in eNOS (eNOS^-/-^) have overwhelmingly shown that these mice experience exacerbated infarct sizes and increased myocardial dysfunction in response to myocardial ischemia [23-26]. In contrast, the overexpression of eNOS has been shown to reduce the size of myocardial infarction and increase myocardial function in the same experimental models of injury [27-29]. Early studies reported that a deficiency of nNOS or iNOS did not affect infarct size in response to acute myocardial ischemia [24,28,30-32]. However, more recent evidence suggests that nNOS plays a crucial role in preventing adverse left ventricular remodeling and ventricular arrhythmias and maintaining myocardial β-adrenergic reserve after myocardial infarction [33,34]. Likewise, new evidence has emerged to suggest that gene transfer of iNOS affords cardioprotection against myocardial I/R injury [35,36]. Taken together, these studies clearly demonstrate that endogenously produced NO has the ability to protect the heart from I/R injury.

Extensive work has also investigated the use of NO as a viable pharmacological approach for the treatment of I/R injury. Inhaled NO gas therapy initiated just before or during coronary artery reperfusion has been shown to be an effective means to rapidly increase the accumulation of NO metabolites in blood and tissues and to provide protection against myocardial I/R injury [37,38]. Additionally, the class of drugs known as NONOates, which release NO in a pH-dependent, first order process have repeatedly been reported to provide cardioprotection in experimental models of myocardial I/R injury [39,40]. NONOates are not the only pharmacological agents that can provide protection by increasing the bioavailability of NO, as it has clearly been shown that statins, metformin, adiponectin, and estrogen provide cardioprotection by increasing the production of NO from eNOS [28,29,41-44]. The use of NO as a therapeutic agent in the treatment of myocardial I/R injury has not been without some controversy, as there have been some studies to report negative effects. In 2001 a comprehensive review investigating the role of NO in modulating myocardial injury spanning from 1991-2001 found that 73% of the studies reported that NO (endogenous or exogenous) was cardioprotective, whereas 12% reported that NO was detrimental [17]. Further investigation of NO efficacy in myocardial I/R have suggested the cause for discrepancies between the opposing findings can be explained by dosing inconsistencies, as it is suggested that physiological levels (i.e., nanomolar) of NO promote cytoprotection, while suprapharmacological levels (i.e. high micromolar and milimolar) mediate cellular necrosis and apoptosis [17,21].

Enhanced expression of HO-1 and its degradation products have been shown to augment multiple intracellular cytoprotective pathways. In particular, HO- 1 protein expression is significantly up-regulated in myocardial infarction [45], and hypoxia-induced upregulation of HO-1 in the heart has been shown to significantly increase CO production [46]. Predictably, studies investigating myocardial damage in HO-1 knockout mice following MI have reported [32] exacerbated myocardial injury, increased ROS production, and decreased endogenous CO production. However, at low levels exogenous CO has been shown to stimulate cardioprotection in HO-1 knockout mice, and rat hearts during I/R [47]. The role of endogenous CO in cardioprotection has also been demonstrated using carbon monoxide-releasing molecules (CO-RMs) to elicit pharmacological activities in myocardial cells against I/R injury [48]. Taken together, these studies suggest the use HO-1 induced CO production and direct administration of CO provide potential therapeutic alternatives for the pharmacological regulation of myocardial I/R injury [9,49,50].

An increasing number of studies also provide evidence that both exogenous and endogenous H_2_S exert cytoprotective effects [51], especially against myocardial I/R injury [52] Studies have found that, targeted deletion and genetic manipulation of CGL leads to modification of H_2_S expression in the aorta, heart, and serum [53]. Johansen first investigated exogenous pre-treatment of H_2_S using a Lagendorff hanging heart model, and found that H_2_S administration caused a reduction in infarct size and suppressed myocardial I/R injury [54]. Similarly, *in vitro *studies have found that pretreatment with H_2_S reduces myocardial necrosis, decreases cardiomyocyte death, improves mitochondrial function [55] and increases myocyte contractility [56,57]. *In vivo *models of myocardial I/R have provided further support suggesting the cardioprotective effects of H_2_S. Studies using murine models I/R injury have shown that of treatment with H_2_S prior to myocardial ischemia significantly reduces infarct size, and H_2_S administered at the time of reperfusion has been shown to reduce infarct size and exert dose dependent cardioprotection [43,58]. However, when the production of H_2_S is reduced by pharmalogical inhibition prior to myocardial ischemia, mice experience exacerbated myocardial injury [58]. Further evidence that H_2_S confers cardioprotection has been shown by genetically altering CGL expression. Mice deficient in CGL (CGL^-/-^) have been reported to experience decreased myocardial function, reduced serum H_2_S levels, pronounced hypertension, diminished endothelium-dependent vasodilation, and significantly larger areas of myocardial infarction compared to wild-type control animals [58-60]. However, a recent study investigating the hemodynamic effects of H2S reported that CGL^-/- ^mice did not display a significant difference in blood pressure when compared to wild-type mice [61]. The discrepancy between these two studies might be partly due to the genetic background of the mice used, which indicates that more research is needed to confirm the effects of CGL inhibition on blood pressure. Furthermore, specific overexpression of CGL has been shown to increase H_2_S production in the heart, and reduce the degree of injury following myocardial I/R [58]. These findings suggest that therapy targeting endogenous and exogenous H_2_S may offer cytoprotection against myocardial I/R injury.

### Other Models of Ischemia-Reperfusion Injury

The cytoprotective effects of gasotransmitter therapy are not limited to myocardial I/R injury, as NO and H_2_S have been shown to confer protection in other organ systems, such as the liver, kidney, and brain. Hepatic I/R injury is oftentimes associated with liver surgery, hepatic transplantation, and hepatic resection. NO modulates hepatocellular/tissue injury through its participation in neutrophil adhesion, platelet aggregation and maintenance of normal vascular permeability [62]. Kuroki et al investigated the role of nitroprusside in the pathogenesis of hepatic I/R injury using a rat model, and reported that it enhances hepatic microcirculation, decreases LDH serum levels and reduces hepatocyte damage [63]. H_2_S therapy has also been shown to reduce serum alanine aminotransferase (ALT) and aspartate aminotransferase (AST) levels following hepatic ischemia-reperfusion [41], and to inhibit lipid peroxidation as well as decrease inflammation [64,65]. In 2007, Tripatara and colleagues used a rat model of renal I/R injury to demonstrate attenuation of renal dysfunction and injury in response to topical treatment with sodium nitrite [66]. In addition, Unal and colleagues [67] have investigated the effects of nitroprusside and antioxidant vitamins C and E, using rat kidney I/R models and found that nitroprusside inhibited xanthine oxidase and provided a preventive influence in renal I/R injury than the antioxidant vitamins C+E. Tripatara and colleagues also investigated the endogenous and exogenous effects of H_2_S in renal I/R injury, and found that CGL inhibition causes a significant decrease in renal function and that topical H_2_S therapy applied to the kidney prior to ischemia improves renal function and attenuates renal I/R injury [68]. More evidence regarding the efficacy of NO and H_2_S therapy has been demonstrated in models of cerebral ischemia. Chen examined the effect of eNOS production in cerebral ischemia using eNOS^-/- ^mice [69]. Predictably, the eNOS^-/- ^mice displayed a significant decrease in neurological function, attenuation of angiogenesis, and decreased cell proliferation. Other studies have reported the benefits of intravenous sodium nitrite infusion at the time of reperfusion as means to restore cerebral blood flow, and decrease infarct volume [70,71]. Furthermore, administration of the exogenous NO donor ZJM-289 has been shown to increase eNOS expression, cGMP, and NO after cerebral ischemia. Moreover, administration of H_2_S following cerebral ischemia has been shown to reduce infarct size, increase H_2_S levels in the brain and provides neuroprotection by inducing hypothermia (30.8 ± 0.7°C) [72]. However, contrasting studies have shown H_2_S administration significantly increases cerebral infarct volume in rats following middle cerebral artery occlusion [73]. A recent study has reported that the neuroprotective effects of H_2_S are concentration dependent [74], and that administration of H_2_S increases fetal GSH levels in the brain, decreases cerebral I/R injury and protects against oxidative stress in utero [75]. Additionally, H_2_S has been shown to reduce neuronal cell death in a murine model of cardiac arrest/cardiopulmonary resuscitation [76]. Thus, additional studies are certainly needed to address the reported discrepancies in models of cerebral injury.

Exposure to CO has been shown to promote cell survival, decrease necrosis, prevent graft rejection and promote tissue protection during organ transplantation [40,77]. Exposure of the graft donor as well as the graft (during ischemia) to exogenous CO and HO-1-derived CO has been shown to restore graft function, reduce generation of ROS and thus prevent cytotoxic tissue injury. Overexpression of HO-1 has been shown to reduce intragraft apoptosis [78] and suppress vascular injury. Akamatsu and colleagues used HO-1 preconditioning to demonstrate retention of functional viability in cardiomyocyte cellular grafts after implantation [79]. Yoshida and colleagues, exposed isolated rat hearts to CO at high pressure, and reported organ preservation, attenuation of intracellular decomposition and prevention of necrosis [80]. Other studies have demonstrated HO-1 increases survival after cardiac transplant and HO-induced CO protects tissue in mouse-to-rat cardiac transplantation [81]. Additionally, at physiological levels CO inhalation was found to exert tissue protection in lung transplantation [82], and HO-1 overexpression has been shown to regulate a cascade of cytoprotective effects in immune response to organ transplantation [83]. Currently the US food and Drug administration has granted an orphan drug safety and tolerability study for CO inhalation therapy in the reduction of delayed graft function, and solid organ transplant preservation.

In summary, extensive research performed in recent years has clearly demonstrated that the efficacy of gasotransmitter therapy in ameliorating *in vitro or in vivo *I/R injury. Most importantly, these studies have provided important information regarding the doses of each gas that provide cytoprotection and suggest that the use of these gases at or near the levels considered to be produced under physiological conditions *in vivo *is optimal to protect a number of organs including the heart, liver, kidney, and brain.

### Summary of Cytoprotective Mechanisms and Evidence for Gasotransmitter Crosstalk

**Figure 4:**
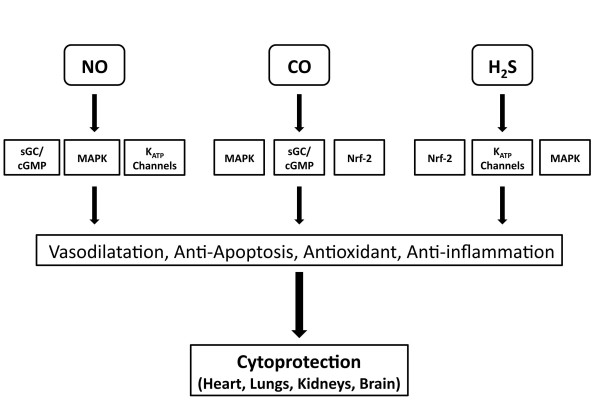
**Summary of Mechanisms by which Gasotransmitters can Induce Cytoprotection**. The gasotransmitters share unique and similar pathways by which they protect against tissue and cellular injury. Both CO and NO have been shown to regulate smooth muscle relaxation through the sGC/cGMP pathway. NO and H_2_S have been shown to regulate cell proliferation and vascular smooth muscle relaxation balance through mitogen-activated protein kinases (MAPK), and ATP-sensitive potassium channels (K_ATP _). In addition, H_2_S and CO regulate oxidant/antioxidant balance through the transcription factor NF-E2-related factor (Nrf2).

So far, this review has provided evidence supporting the multifaceted role of gasotransmitters in cytoprotection and as such has highlighted the similarities between all three gases. For instance, all are naturally produced in the body and are constantly participating in biological responses within target tissues and organs [84]. The rate of NO/CO/H_2_S production, cytoprotection and clearance vary with time, dose concentration, and enzymatic mediators. Even the enzymes responsible for biosynthesis of gastrotransmitters show parallel similarities and in the case of NO and CO can be classified as constitutive (eNOS, nNOS, and HO-2) or inducible (iNOS, and HO1). Importantly, all three gasotransmitters possess similar physiological actions that could account for the observed cytoprotective effects (Figure 4). For example, all three can: (1) induce vasodilatation by activating the sGC/cGMP pathway (NO and CO) [85] or by activating ATP-sensitive K^+ ^(K_ATP_) channels (H_2_S) [15]; (2) inhibit apoptosis by directly interacting with the apoptotic machinery [86] or by increasing the expression of anti-apoptogens, such as HSP90, HSP70, and Bcl-2 [43]; (3) modulate mitochondrial respiration [17,58,87]; (4) induce antioxidants [58,81] and (5) inhibit inflammation [88-91]. However, while the actions are similar, there are some differences in the mechanisms by which these gasotransmitters induce these effects and the regulatory actions of the enzyme systems can vary depending upon the gas being investigated. Furthermore, there does appear to be some crosstalk between the gases, which can provide synergistic effects and additional regulatory effects. The rest of this article will provide a brief discussion on the complex interactions between the gasotransmitter systems.

We will begin with the interaction between NO and H_2_S. Like NO, H_2_S is produced in the endothelium as well as SMCs [92], and mediates acute regulation by vasorelaxative hormones through calmodulin and IP_3 _dependent pathways [93]. There appears to be a close interaction between H_2_S and NO, with NO amplifying the inhibitory effect of H_2_S and H_2_S tissue specific activation of eNOS [94]. In particular, NO and H_2_S have been suggested to collaborate in regulating vascular homeostasis and vasodilation [14]. Additional evidence suggests that NO can increase CGL activity acutely, and that chronic exposure to NO up-regulates CGL expression. Moreover, at low concentrations H_2_S has been shown to enhance the release of NO from vascular endothelium and increase the vasorelaxant effect of the NO donor sodium nitroprusside [15].

CO and NO share apparent similarities in structure, molecular weight and solubility [95]. Both NO and CO interact with iron (Fe) to form 5 or 6 coordinated haem complexes, which result in conformational changes and activation of the sGC/cGMP pathway [85]. Thus, many of the biological effects of CO are similar to NO, including its anti-apoptotic, anti-proliferative and anti-inflammatory mechanisms. Other studies have confirmed the participation of both NO and CO-mediated signaling cascades in immune suppression of platelet aggregation and neurotransmission [96]. In addition to regulating vascular cell growth, CO influences cell survival by blocking cytokine-mediated mitochondrial release of cytochrome *C *[97] and has been shown to influence hepatoprotection through the transcriptional upregulation of iNOS in the liver. Both exogenously administered or endogenously released NO stimulates HO-1 gene expression and CO production [32,97]. Furthermore, CO and NO have been shown to participate in vasoactive cross talk, influencing: growth factors, anti-inflammatory mediators, angiogenesis and vascular remodeling [98,99].

The crosstalk between H_2_S and CO has been the least studied, as there are only a few studies which have addressed this interactions. Zhang and colleagues [100] were one of the first to investigate the physiological and pathological interactions of CO and H_2_S and found that exogenous H_2_S can upregulate the CO/HO pathway during hypoxic pulmonary hypertension. Additionally, in much the same manner to that described for NO, H_2_S increases the expression of HO-1 in a Nrf-2 dependent manner [43].

## Conclusion

The studies mentioned in this review have identified the therapeutic potential and translational opportunities of gasotransmitters as potent cytoprotective molecules. So far, the exogenous administration, endogenous manipulation and use of genetically modified animals has been successful in demonstrating gasotransmitter-mediated cytoprotection in models of I/R injury and other forms of disease. In addition, the gasotransmitters have been shown to play a pivotal role in the regulation of cell functions and in the reduction of tissue injury by activation of a number of prosurvival pathways. However, there are still a number of questions that remain to be answered, especially in relation to the interactions between the gases. For instance, the exact correlation between these gases in the various pathways of cytoprotection has yet to be fully investigated [101]. It is also not known if using some variation of NO/CO/H_2_S, as a combination therapy will provide synergistic effects in the treatment of ischemic disorders. Therefore, additional studies designed to examine NO/CO/H_2_S cross talk will provide better comprehension concerning this issue, as well as new insights into their interactions. In addition, it is important to recognize a need for the development of consistent dosing and measurement techniques for the advancement of gasotransmitters in pharmalogical research. Because the regulation, expression and function of these gaseous molecules are so complex, optimal alterations in synthesis and activity will possibly provide novel therapeutic opportunities for the treatment of a number of pathophysiological conditions.

## List of Abbreviations

3MST: 3-metacaptopyruvate sulfur transferase; ALT: alanine aminotransferase; AST: aspartate aminotransferase; Bcl-2: B-cell lymphoma 2; CBS: cystathionine β-synthase; CGL: cystathionine γ-lyase; cGMP: cyclic guanosine monophosphate; CNS: central nervous system; CO: carbon monoxide; CO-RM: carbon monoxide -releasing molecules; eNOS: endothelial nitric oxide synthase; Fe: iron; GC: guanylyl cylase; H_2_S: hydrogen sulfide; HO: heme oxygenase; HSP: heat shock protein; I/R: ischemia reperfusion; iNOS: inducible nitric oxide synthase; IP3: inositol trisphosphate; K_ATP_: ATP-sensitive K^+ ^channels; MI/R: myocardial ischemia reperfusion; NADPH: nicotinamide adenine dinucleotide phosphate-oxidase; NANC: nonadrenergic/noncholinergic; nNOS: neuronal nitric oxide synthase; NO: nitric oxide; NOS: nitric oxide synthase; Nrf2: nuclear factor-E2-related factor-2; SMC: smooth muscle cell.

## Competing interests

The authors declare that they have no competing interests.

## Authors' contributions

BFM and JWC wrote the manuscript. All authors have read and approved the final manuscript.

## References

[B1] Nicholson CK Calvert JW Hydrogen sulfide and ischemia-reperfusion injury Pharmacol Res 2010 62 289 297 10.1016/j.phrs.2010.06.002 20542117 PMC2917489

[B2] Szabo C Ischiropoulos H Radi R Peroxynitrite: biochemistry, pathophysiology and development of therapeutics Nat Rev Drug Discov 2007 6 662 680 10.1038/nrd2222 17667957

[B3] Brunton TL On the use of nitrite of amyl in angina pectoris 1867 2

[B4] Furchgott RF Zawadzki JV The obligatory role dof endothelial cells in the relazation of arterial smooth muscle by acetylcholine 1980 288 288 373 10.1038/288373a0 6253831

[B5] Choi AM Otterbein LE Emerging role of carbon monoxide in physiologic and pathophysiologic states Antioxid Redox Signal 2002 4 227 228 10.1089/152308602753666271 12006173

[B6] Wang R Resurgence of carbon monoxide: an endogenous gaseous vasorelaxing factor Can J Physiol Pharmacol 1998 76 1 15 10.1139/y97-187 9564544

[B7] Verma A Hirsch D Glatt C Ronett G Snyder S Carbon monoxide: A putative neural messenger Science 1993 259 381 384 10.1126/science.7678352 7678352

[B8] Ryter SW Choi AM Heme oxygenase-1: molecular mechanisms of gene expression in oxygen-related stress Antioxid Redox Signal 2002 4 625 632 10.1089/15230860260220120 12230874

[B9] Perrella MA Yet SF Role of heme oxygenase-1 in cardiovascular function Curr Pharm Des 2003 9 2479 2487 10.2174/1381612033453776 14529547

[B10] Marshall HE Stamler JS Exhaled nitric oxide (NO), NO synthase activity, and regulation of nuclear factor (NF)-kappaB Am J Respir Cell Mol Biol 1999 21 296 297 10460745 10.1165/ajrcmb.21.3.f164

[B11] Foresti R Motterlini R The heme oxygenase pathway and its interaction with nitric oxide in the control of cellular homeostasis Free Radic Res 1999 31 459 475 10.1080/10715769900301031 10630670

[B12] Kimura H Hydrogen sulfide: its production, release and functions J Neurosci 1996 16 1066 10718558235

[B13] Shibuya N Tanaka M Yoshida M Ogasawara Y Togawa T Ishii K Kimura H 3-Mercaptopyruvate sulfurtransferase produces hydrogen sulfide and bound sulfane sulfur in the brain Antioxid Redox Signal 2009 11 703 714 10.1089/ars.2008.2253 18855522

[B14] Hosoki R Matsuki N Kimura H The possible role of hydrogen sulfide as an endogenous smooth muscle relaxant in synergy with nitric oxide Biochem Biophys Res Commun 1997 237 527 531 10.1006/bbrc.1997.6878 9299397

[B15] Zhao W Zhang J Lu Y Wang R The vasorelaxant effect of H(2)S as a novel endogenous gaseous K(ATP) channel opener EMBO J 2001 20 6008 6016 10.1093/emboj/20.21.6008 11689441 PMC125693

[B16] Wang R The gasotransmitter role of hydrogen sulfide 2003 5 493 501 10.1089/152308603768295249 13678538

[B17] Bolli R Cardioprotective function of inducible nitric oxide synthase and role of nitric oxide in myocardial ischemia and preconditioning: an overview of a decade of research J Mol Cell Cardiol 2001 33 1897 1918 10.1006/jmcc.2001.1462 11708836

[B18] Jugdutt B Nitric oxide and cardiovascular protection Heart Fail Rev 2003 8 29 34 10.1023/A:1022190820131 12652157

[B19] Ferdinandy P Schulz R Nitric oxide, superoxide and peroxynitrite inmyocardial ischaemia-reperfusion injury and preconditioning Br J Pharmacol 2003 138 532 543 10.1038/sj.bjp.0705080 12598407 PMC1573696

[B20] Schulz R Kelm M Heusch G Nitric oxide in myocardial ischemia/reperfusion injury Cardiovasc Res 2004 61 402 413 10.1016/j.cardiores.2003.09.019 14962472

[B21] Jones SP Bolli R The ubiquitous role of nitric oxide in cardioprotection 2006 40 16 23 10.1016/j.yjmcc.2005.09.011 16288777

[B22] Bannenberg G Vieira H Therapeutic applications of the gaseous mediators carbon monoxide and hydrogen sulfide Expert Opin Ther Pat 2009 19 663 682 10.1517/13543770902858824 19441940

[B23] Hannan RL John MC Kouretas PC Hack BD Matherne GP Laubach VE Deletion of endothelial nitric oxide synthase exacerbates myocardial stunning in an isolated mouse heart model J Surg Res 2000 93 127 132 10.1006/jsre.2000.5953 10945953

[B24] Sumeray MS Rees DD Yellon DM Infarct size and nitric oxide synthase in murine myocardium J Mol Cell Cardiol 2000 32 35 42 10.1006/jmcc.1999.1050 10652188

[B25] Sharp BR Jones SP Rimmer DM Lefer DJ Differential response to myocardial reperfusion injury in eNOS-deficient mice Am J Physiol Heart CIrc Physiol 2002 282 H2422 2426 12003854 10.1152/ajpheart.00855.2001

[B26] Jones SP Girod WG Palazzo AJ Granger DN Grisham MB Jourd'Heuil D Huang PL Lefer DJ Myocardial ischemia-reperfusion injury is exacerbated in absence of endothelial cell nitric oxide synthase Am J Physiol 1999 276 H1567 1573 10330240 10.1152/ajpheart.1999.276.5.H1567

[B27] Jones AM Wilkerson DP Koppo K Wilmshurst S Campbell IT Inhibition of nitric oxide synthase by L-NAME speeds phase II pulmonary. VO2 kinetics in the transition to moderate-intensity exercise in man J Physiol 2003 552 265 272 10.1113/jphysiol.2003.045799 12897178 PMC2343309

[B28] Jones SP Lefer DJ Myocardial Reperfusion Injury: Insights Gained from Gene-Targeted Mice News Physiol Sci 2000 15 303 308 11390931 10.1152/physiologyonline.2000.15.6.303

[B29] Elrod JW Greer JJ Bryan NS Langston W Szot JF Gebregzlabher H Janssens S Feelisch M Lefer DJ Cardiomyocyte-specific overexpression of NO synthase-3 protects against myocardial ischemia-reperfusion injury Arterioscler Thromb Vasc Biol 2006 26 1517 1523 10.1161/01.ATV.0000224324.52466.e6 16645153

[B30] Jones S Greer J Kakkar A Ware P Turnage R Hicks M vanHaperen R de Crom R Kawashima S Yokoyama M Lefer D Endothelial nitric oxide synthatse overexpression attenuates myocardial reperfusion injury Am J Physiol Heart Cir Physiol 2004 286 H276 282 10.1152/ajpheart.00129.2003 12969888

[B31] Xi L Nitric oxide-dependent mechanism of anti-ischemic myocardial protection induced by monophosphoryl lipid A Zhongguo Yao Li Xue Bao 1999 20 865 871 11270982

[B32] Liu X Chapman G Peyton K Schafer A Durante W Carbon monoxide inhibits apoptosis in vascular smooth muscle cells Cardiovasc Res 2002 55 396 405 10.1016/S0008-6363(02)00410-8 12123779

[B33] Burger DE Xiang FL Hammoud L Jones DL Feng Q Erythropoietin protects the heart from ventricular arrhythmia during ischemia and reperfusion via neuronal nitric-oxide synthase J Pharmacol Exp Ther 2009 329 900 907 10.1124/jpet.109.150896 19307451

[B34] Dawson D Lygate CA Zhang MH Hulbert K Neubauer S Casadei B nNOS gene deletion exacerbates pathological left ventricular remodeling and functional deterioration after myocardial infarction Circulation 2005 112 3729 3737 10.1161/CIRCULATIONAHA.105.539437 16344403

[B35] Li T Li J Liu J Zhang P Wu W Zhou R Li G Zhang W Yi M Huang H Polymerized placenta hemoglobin attenuates ischemia/reperfusion injury and restores the nitroso-redox balance in isolated rat heart Free Radic Biol Med 2009 46 397 405 10.1016/j.freeradbiomed.2008.10.042 19038330

[B36] Li Q Guo Y Tan W Ou Q Wu WJ Sturza D Dawn B Hunt G Cui C Bolli R Cardioprotection afforded by inducible nitric oxide synthase gene therapy is mediated by cyclooxygenase-2 via a nuclear factor-kappaB dependent pathway Circulation 2007 116 1577 1584 10.1161/CIRCULATIONAHA.107.689810 17785622 PMC3654387

[B37] Yamashita H Akamin S Sumida Y Inoue M Sawada T Nagayasu T Oka T Inhaled nitric oxide attenuates apoptosis in ishemia-reperfusion injury of rabbit lungs The annals of thoracic surgery 78 292 297 204 10.1016/j.athoracsur.2003.12.025 15223447

[B38] Liu X Huang Y Pokreisz P Vermeersch P Marsboom G Swinnen M Verbeken E Santos J Pellens M Gillijns H Nitric oxide inhalation improves microvascular flow and decreases infarction size after myocardial ischemia and reperfusion J Am Coll Cardiol 2007 50 808 817 10.1016/j.jacc.2007.04.069 17707188

[B39] Takano H Manchikalapudi S Tang XL Qiu Y Rizvi A Jadoon AK Zhang Q Bolli R Nitric oxide synthase is the mediator of late preconditioning against myocardial infarction in conscious rabbits Circulation 1998 98 441 449 9714095 10.1161/01.cir.98.5.441

[B40] Wang G Liem DA Vondriska TM Honda HM Korge P Pantaleon DM Qiao X Wang Y Weiss JN Ping P Nitric oxide donors protect murine myocardium against infarction via modulation of mitochondrial permeability transition Am J Physiol Heart CIrc Physiol 2005 288 H1290 1295 15528225 10.1152/ajpheart.00796.2004

[B41] Jha S Calvert J Duranski M Ramachandran A Lefer D Hydrogen sulfide attenuates hepatic ischemia-reperfusion injury: role of antioxidant and antiapoptotic signaling Am J Physiol Heart Circ Physiol 2008 295 H801 806 10.1152/ajpheart.00377.2008 18567706 PMC2519205

[B42] Li Q Sun B Wang X Jin Z Zhou Y Dong L Jiang LH Rong W A crucial role for hydrogen sulfide in oxygen sensing via modulating large conductance calcium-activated potassium channels Antioxid Redox Signal 2010 12 1179 1189 10.1089/ars.2009.2926 19803741

[B43] Calvert JW Jha S Gundewar S Elrod JW Ramachandran A Pattillo CB Kevil CG Lefer DJ Hydrogen sulfide mediates cardioprotection through Nrf2 signaling Circ Res 2009 105 365 374 10.1161/CIRCRESAHA.109.199919 19608979 PMC2735849

[B44] Jones NC Constantin D Gibson CL Prior MJ Morris PG Marsden CA Murphy S A detrimental role for nitric oxide synthase-2 in the pathology resulting from acute cerebral injury J Neuropathol Exp Neurol 2004 63 708 720 15290896 10.1093/jnen/63.7.708

[B45] Lakkisto P Palojoki E Backlund T Saraste A Tikkanen I Voipio-Pulkki LM Pulkki K Expression of heme oxygenase-1 in response to myocardial infarction in rats J Mol Cell Cardiol 2002 34 1357 1365 10.1006/jmcc.2002.2094 12392996

[B46] Grilli A De Lutiis MA Patruno A Speranza L Gizzi F Taccardi AA Di Napoli P De Caterina R Conti P Felaco M Inducible nitric oxide synthase and heme oxygenase-1 in rat heart: direct effect of chronic exposure to hypoxia Ann Clin Lab Sci 2003 33 208 215 12817626

[B47] Mei DS Du YA Wang Y Cardioprotection and mechanisms of exogenous carbon monoxide releaser CORM-2 against ischemia/reperfusion injury in isolated rat hearts Zhejiang Da Xue Xue Bao Yi Xue Ban 2007 36 291 297 17571314 10.3785/j.issn.1008-9292.2007.03.014

[B48] Clark JE Kottam A Motterlini R Marber MS Measuring left ventricular function in the normal, infarcted and CORM-3-preconditioned mouse heart using complex admittance-derived pressure volume loops J Pharmacol Toxicol Methods 2009 59 94 99 10.1016/j.vascn.2008.10.007 19059354

[B49] Yoshida J Ozaki K Nalesnik M Ueki S Castillo-Rama M Faleo G Ezzelarab M Nakao A Ekser B Echeverri G Ex vivo application of carbon monoxide in UW solutionprevents transplant-induced renal ischemia/reperfusion injury in pigs Am J Transplant 2010 10 763 772 10.1111/j.1600-6143.2010.03040.x 20199500 PMC2886983

[B50] Guo Y Stein A Wu W Tan W Zhu X Li Q Dawn B Motterlini R Bolli R Administration of a Co-releasing molecule at the time of reperfusion reduces infarct size in vivo Am J Physiol Heart Circ Physiol 2004 286 H1649 1653 10.1152/ajpheart.00971.2003 14704226 PMC3208268

[B51] Kimura Y Kimura H Hydrogen sulfide protects neurons from oxidative stress FASEB J 2004 18 1165 1167 15155563 10.1096/fj.04-1815fje

[B52] Mancardi D Penna C Merlino A Del Soldato P Wink DA Pagliaro P Physiological and pharmacological features of the novel gasotransmitter: hydrogen sulfide Biochim Biophys Acta 2009 1787 864 872 10.1016/j.bbabio.2009.03.005 19285949 PMC3538351

[B53] Mustafa AK Gadalla MM Snyder SH Signaling by gasotransmitters Sci Signal 2009 2 re2 10.1126/scisignal.268re2 19401594 PMC2744355

[B54] Johansen D Ytrehus K Baxter GF Exogenous hydrogen sulfide (H2S) protects against regional myocardial ischemia-reperfusion injury--Evidence for a role of K ATP channels Basic Res Cardiol 2006 101 53 60 10.1007/s00395-005-0569-9 16328106

[B55] Elsey DJ Fowkes RC Baxter GF Regulation of cardiovascular cell function by hydrogen sulfide (H(2)S) Cell Biochem Funct 2010 28 95 106 10.1002/cbf.1618 20104507

[B56] Hu Y Chen X Pan T Neo K Lee S Khin E Moore P Bian J Cardioprotection induced by hydrogen sulfide preconditioning involves activation of ERK and PI3K/Akt pathways Pflugers Arch 2008 455 607 616 17674030 10.1007/s00424-007-0321-4

[B57] Bian JS Yong Q Pan T Feng Z Ali M Zhou S Moore P Role of hydrogen sulfide in the cardioprotection caused by ischemic preconditioning in the rat heart and cardiac myocytes J Pharmacol Exp Ther 2006 316 670 678 16204473 10.1124/jpet.105.092023

[B58] Elrod JW Calvert JW Morrison J Doeller JE Kraus DW Tao L Jiao X Scalia R Kiss L Szabo C Hydrogen sulfide attenuates myocardial ischemia-reperfusion injury by preservation of mitochondrial function Proc Natl Acad Sci USA 2007 104 15560 15565 10.1073/pnas.0705891104 17878306 PMC2000503

[B59] Sivarajah A Collino M Yasin M Benetti E Gallicchio M Mazzon E Cuzzocrea S Fantozzi R Thiemermann C Anti-apoptotic and anti-inflammatory effects of hydrogen sulfide in a rat model of regional myocardial I/R Shock 2009 31 267 274 10.1097/SHK.0b013e318180ff89 18636044

[B60] Yang G Wu L Jiang B Yang W Qi J Cao K Meng Q Mustafa AK Mu W Zhang S H2S as a physiologic vasorelaxant: hypertension in mice with deletion of cystathionine gamma-lyase Science 2008 322 587 590 10.1126/science.1162667 18948540 PMC2749494

[B61] Ishii I Akahoshi N Yamada H Nakano S Izumi T Suematsu M Cystathionine gamma-Lyase-deficient mice require dietary cysteine to protect against acute lethal myopathy and oxidative injury J Biol Chem 2010 285 26358 26368 10.1074/jbc.M110.147439 20566639 PMC2924062

[B62] Phillips L Lopez-Nebllna F Toledo A Anaya-Prado R Toledo-Pereyra p Nitric Oxide Mechanism of protection in ischemia reperfusion injury Invs Surgery 2009 22 46 55 10.1080/08941930802709470 19191157

[B63] Kuroki I Miyazaki T Mizukami I Matsumoto N Matsumoto I Effect of sodium nitroprusside on ischemia-reperfusion injuries of the rat liver Hepatogastroenterology 2004 51 1404 1407 15362764

[B64] Kang K Zhao M Jiang H Tan G Pan S Sun X Role of hydrogen sulfide in hepatic ischemia-reperfusion-induced injury in rats Liver Transpl 2009 15 1306 1314 10.1002/lt.21810 19790158

[B65] Xu GY Winston JH Shenoy M Zhou S Chen JD Pasricha PJ The endogenous hydrogen sulfide producing enzyme cystathionine-beta synthase contributes to visceral hypersensitivity in a rat model of irritable bowel syndrome Mol Pain 2009 5 44 10.1186/1744-8069-5-44 19660142 PMC2731739

[B66] Tripatara P Patel NS Webb A Rathod K Lecomte FM Mazzon E Cuzzocrea S Yaqoob MM Ahluwalia A Thiemermann C Nitrite-derived nitric oxide protects the rat kidney against ischemia/reperfusion injury in vivo: role for xanthine oxidoreductase J Am Soc Nephrol 2007 18 570 580 10.1681/ASN.2006050450 17202421

[B67] Unal D Yeni E Erel O Bitiren M Vural H Antioxidative effects of exogenous nitric oxide versus antioxidant vitamins on renal ischemia reperfusion injury Urol Res 2002 30 190 194 10.1007/s00240-002-0254-5 12111183

[B68] Tripatara P Patel NS Collino M Gallicchio M Kieswich J Castiglia S Benetti E Stewart KN Brown PA Yaqoob MM Generation of endogenous hydrogen sulfide by cystathionine gamma-lyase limits renal ischemia/reperfusion injury and dysfunction Lab Invest 2008 88 1038 1048 10.1038/labinvest.2008.73 18679378

[B69] Chen SH Cheung RT Neuropeptide Y and its receptor analogs differentially modulate the immunoreactivity for neuronal or endothelial nitric oxide synthase in the rat brain following focal ischemia with reperfusion J Biomed Sci 2005 12 267 278 10.1007/s11373-005-1359-y 15942706

[B70] Xing Y Xu ZX Mang J Qian JL Vascular endothelial growth factor expression in focal cerebral ischemia/reperfusion in injury in rats Zhongguo Wei Zhong Bing Ji Jiu Yi Xue 2005 17 174 176 15760533

[B71] Calvert JW Lefer DJ Myocardial protection by nitrite Cardiovasc Res 2009 83 195 203 10.1093/cvr/cvp079 19251721 PMC2701719

[B72] Florian B Vintilescu R Balseanu AT Buga AM Grisk O Walker LC Kessler C Popa-Wagner A Long-term hypothermia reduces infarct volume in aged rats after focal ischemia Neurosci Lett 2008 438 180 185 10.1016/j.neulet.2008.04.020 18456407

[B73] Qu K Chen CP Halliwell B Moore PK Wong PT Hydrogen sulfide is a mediator of cerebral ischemic damage Stroke 2006 37 889 893 10.1161/01.STR.0000204184.34946.41 16439695

[B74] Ren G Bardwell J Engineered pathways for correct disulfide bond oxidation Antioxid Redox Signal in press 10.1089/ars.2010.3782 PMC3096521 21250836

[B75] Kimura Y Goto Y Kimura H Hydrogen sulfide increases glutathione production and suppresses oxidative stress in mitochondria Antioxid Redox Signal 2010 12 1 13 10.1089/ars.2008.2282 19852698

[B76] Minamishima S Bougaki M Sips PY Yu JD Minamishima YA Elrod JW Lefer DJ Bloch KD Ichinose F Hydrogen sulfide improves survival after cardiac arrest and cardiopulmonary resuscitation via a nitric oxide synthase 3-dependent mechanism in mice Circulation 2009 120 888 896 10.1161/CIRCULATIONAHA.108.833491 19704099 PMC2768477

[B77] Ryter S Morse D Choi A Heme oxygenase-1/carbon monoxide:from basic science to therapeutic applications Antioxid Red Siganl 2010 12 1 13 10.1089/ars.2008.2282

[B78] Katori M Busuttil RW Kupiec-Weglinski JW Heme oxygenase-1 system in organ transplantation Transplantation 2002 74 905 912 10.1097/00007890-200210150-00001 12394829

[B79] Akamatsu Y Haga M Tyagi S Yamashita K Graca-Souza AV Ollinger R Czismadia E May GA Ifedigbo E Otterbein LE Heme oxygenase-1-derived carbon monoxide protects hearts from transplant associated ischemia reperfusion injury FASEB J 2004 18 771 772 14977880 10.1096/fj.03-0921fje

[B80] Yoshida Y Hatayama N Seki K Study on the preservation with CO (PCO = 200-2,000 hPa), resuscitation, and heterotopic transplantation of an isolated rat heart Cell Transplant 2009 18 535 540 19775514 10.1177/096368970901805-608

[B81] Sato K Balla J Otterbein L Smith RN Brouard S Lin Y Csizmadia E Sevigny J Robson SC Vercellotti G Carbon monoxide generated by heme oxygenase-1 suppresses the rejection of mouse-to-rat cardiac transplants J Immunol 2001 166 4185 4194 11238670 10.4049/jimmunol.166.6.4185

[B82] Zhou HC Ding WG Cui XG Pan P Zhang B Li WZ Carbon monoxide inhalation ameliorates conditions of lung grafts from rat brain death donors Chin Med J (Engl) 2008 121 1411 1419 18959119

[B83] Williams JW Mital D Chong A Kottayil A Millis M Longstreth J Huang W Brady L Jensik S Experiences with leflunomide in solid organ transplantation Transplantation 2002 73 358 366 10.1097/00007890-200202150-00008 11884931

[B84] Hartsfield CL Cross talk between carbon monoxide and nitric oxide Antioxid Redox Signal 2002 4 301 307 10.1089/152308602753666352 12006181

[B85] Boehning D Snyder S Novel neural modulators Ann Rev Neurosci 2003 26 105 131 10.1146/annurev.neuro.26.041002.131047 14527267

[B86] Maejima Y Adachi S Morikawa K Ito H Isobe M Nitric oxide inhibits myocardial apoptosis by preventing caspase-3 activity via S-nitrosylation J Mol Cell Cardiol 2005 38 163 174 10.1016/j.yjmcc.2004.10.012 15623433

[B87] Rakhit R Mojet M Marber M Duchen M Mitochondria as targets for nitric oxide-induced protection during simulated ischemia and reoxygenation in isolated neonatal cardiomyocytes Ciruculation 2001 103 2617 2623 10.1161/01.cir.103.21.2617 11382733

[B88] Korhonen R Lahti A Kankaanranta H Moilanen E Nitric oxide production and signaling in inflammation Curr Drug Targets Inflamm Allergy 2005 4 471 479 10.2174/1568010054526359 16101524

[B89] Wang R Wang Z Wu L Carbon monoxide-induced vasorelaxation and the underlying mechanisms Br J Pharmacol 1997 121 927 934 10.1038/sj.bjp.0701222 9222549 PMC1564776

[B90] Otterbein L The evolution of carbon monoxide into medicine Respir Care 2009 54 925 932 10.4187/002013209793800394 19558742

[B91] Zhou H Liu J Pan P Jin D Ding W Li W Carbon monoxide inhalation decreased lung injury via anti-inflammatory and anti-apoptotic effects in brain death rats Exp Biol Med (Maywood) 2010 235 1236 1243 10.1258/ebm.2010.010147 20810760

[B92] Shibuya N Mikami Y Kimura Y Nagahara N Kimura H Vascular endothelium expresses 3-mercaptopyruvate sulfurtransferase and produces hydrogen sulfide J Biochem 2009 146 623 626 10.1093/jb/mvp111 19605461

[B93] Wagner CA Hydrogen sulfide: a new gaseous signal molecule and blood pressure regulator J Nephrol 2009 22 173 176 19384833

[B94] Kasparek MS Linden DR Kreis ME Sarr MG Gasotransmitters in the gastrointestinal tract Surgery 2008 143 455 459 10.1016/j.surg.2007.10.017 18374039 PMC2440668

[B95] Ryter SW Morse D Choi AM Carbon monoxide: to boldly go where NO has gone before Sci STKE 2004 2004 RE6 10.1126/stke.2302004re6 15114002

[B96] Nathan C Nitric oxide as a secretory product of mammalian cells FASEB J 1992 6 3051 3064 1381691

[B97] Durante W Targeting heme oxygenase-1 in vascular disease Curr Drug Targets 2010 11 1504 1516 20704550 10.2174/1389450111009011504 PMC2978667

[B98] Li XH Du JB Bu DF Tang XY Tang CS Sodium hydrosulfide alleviated pulmonary vascular structural remodeling induced by high pulmonary blood flow in rats Acta Pharmacol Sin 2006 27 971 980 10.1111/j.1745-7254.2006.00353.x 16867247

[B99] Wanstall J Jeffery T Gambino A Lovren F Triggle C Vascular smooth muscle relaxation mediated by nitric oxide donors: a comparison with acetylcholine, nitric oxide and nitroxyl ion Br J Pharmaco 2001 134 463 472 10.1038/sj.bjp.0704269 PMC1572971 11588100

[B100] Zhang QY Du JB Zhang CY Tang CS The regulation of carbon monoxide/heme oxygenase system by hydrogen sulfide in rats with hypoxic pulmonary hypertension Zhonghua Jie He He Hu Xi Za Zhi 2004 27 659 663 16200866

[B101] Burger D Xiang H Lu X Feng Q Role of heme oxygenase-1 in the cardioprotective effects of erythorpoietin during myocardial ischemia Am J Physiol Heart Circ Physiol 2009 1 H84 93 10.1152/ajpheart.00372.2008 18996987

